# Disruption of the ammonium transporter *AMT1.1* alters basal defenses generating resistance against *Pseudomonas syringae* and *Plectosphaerella cucumerina*

**DOI:** 10.3389/fpls.2014.00231

**Published:** 2014-05-30

**Authors:** Victoria Pastor, Jordi Gamir, Gemma Camañes, Miguel Cerezo, Paloma Sánchez-Bel, Victor Flors

**Affiliations:** ^1^Laboratoire de Biologie Moleculaire et Cellulaire, Faculté des Sciences, Institut de Biologie, Universite de NeuchatelNeuchatel, Switzerland; ^2^Área de Fisiología Vegetal, Departamento de Ciencias Agrarias y del Medio Natural, Universitat Jaume ICastellón, Spain

**Keywords:** *AMT1.1*, basal resistance, metabolomics, transceptor, *NRT2.1*

## Abstract

Disruption of the high-affinity nitrate transporter *NRT2.1* activates the priming defense against *Pseudomonas syringae*, resulting in enhanced resistance. In this study, it is demonstrated that the high-affinity ammonium transporter *AMT1.1* is a negative regulator of *Arabidopsis* defense responses. The T-DNA knockout mutant *amt1.1* displays enhanced resistance against *Plectosphaerella cucumerina* and reduced susceptibility to *P. syringae*. The impairment of *AMT1.1* induces significant metabolic changes in the absence of challenge, suggesting that *amt1.1* retains constitutive defense responses. Interestingly, *amt1.1* combats pathogens differently depending on the lifestyle of the pathogen. In addition, N starvation enhances the susceptibility of wild type plants and the mutant *amt1.1* to *P. syringae* whereas it has no effect on *P. cucumerina* resistance. The metabolic changes of *amt1.1* against *P. syringae* are subtler and are restricted to the phenylpropanoid pathway, which correlates with its reduced susceptibility. By contrast, the *amt1.1* mutant responds by activating higher levels of camalexin and callose against *P. cucumerina*. In addition, *amt1.1* shows altered levels of aliphatic and indolic glucosinolates and other Trp-related compounds following infection by the necrotroph. These observations indicate that *AMT1.1* may play additional roles that affect N uptake and plant immune responses.

## Introduction

*Arabidopsis* defense responses against *P. syringae* and *P. cucumerina* have been widely studied. These pathogens have very different life styles, in that *P. syringae* is a hemibiotrophic bacterial pathogen that penetrates through natural openings, whereas *P. cucumerina* is a necrotrophic fungus that can overcome cell barriers and penetrate into the cytoplasm (Berrocal-Lobo et al., 2002). Salicylic acid-dependent defenses have emerged as the most effective resistance mechanism against the bacterium (Brooks et al., 2005; Glazebrook, 2005). The effector secretion machinery of *P. syringae* is currently under study because of its effectiveness in suppressing *Arabidopsis* defenses. Although the direct link remains unknown, Camañes et al. (2012b) demonstrated that coronatine, a *P. syringae* effector, targets *NRT2.1* and subsequently causes a H_2_O_2_ burst. The *nrt2* mutant remains insensitive to this manipulation and the increased JA-signaling that is responsive to coronatine (Melotto et al., 2006). *P. cucumerina* is more complex. *Arabidopsis* defenses against this pathogen are horizontal and involve several defense signaling pathways, such as SA, JA, IAA, and ABA-mediated callose deposition (Lorenzo and Solano, 2005; García-Andrade et al., 2011; Sánchez-Vallet et al., 2012; Gamir et al., 2014). N fertilization influences plant-pathogen interactions; therefore, the following two factors affect plant resistance: the total amount of N and the source of N. Over-fertilization increases the severity of mildew in several grapevine cultivars and many crop plants (Keller et al., 2003; Marschner, 2012). The level of N in fertilization programs also affects plant resistance based on the pathogen lifestyle. For example, elevated levels of N promote susceptibility against biotrophs, such as powdery mildew, whereas it results in reduced disease development of *Botrytis cinerea* (Walters and Bingham, 2007). However, the source of N appears to have a greater influence in plant resistance (Gupta et al., 2013). Fertilizing tobacco plants with NO^−^_3_ accelerates the hypersensitive response following *P. syringae* pv. *Phaseolicola* infections and provides enhanced resistance. This resistance is related to NO^−^_3_ reduction and NO production that triggers SA synthesis (Durner and Klessig, 1999). Plants fed NO^−^_3_ display increased *PR1* expression and higher levels of SA. By contrast, tobacco plants fed with NH^+^_4_ are more susceptible to the bacterium. The bypass of nitrate reduction avoids NO production and triggers 4-aminobutyric accumulation, which is a nutrient for the pathogen (Solomon and Oliver, 2001), increasing the susceptibility of the plant.

Previously, first in animal sciences and then in plant sciences, the term transceptor is applied to membrane proteins that perform a dual transport and signaling function. Gojon et al. (2011) revised the transceptor role of the nitrate transporter family member NRT1. In addition to binding to low affinity nitrate, this protein can sense nitrate in the root environment. Additional studies demonstrated that both NRT1.1 and the high-affinity transporter NRT2.1 are transceptors. The sensing network regulated by NRT1.1 has not yet been elucidated, although several of its components have been recently described. One target of NRT1.1 is the transcription of *NRT2.1*. The dual role of the induction of *NRT2.1* during early time-points of nitrate supply and repression during later time-points both directly and indirectly involve NRT1.1. Of the N metabolism components, mutants impaired in *NRT1.1*, such as *chl1* are also affected in *NIA1, NiR* (encoding nitrate reductase and nitrite reductase, respectively) (Wang et al., 2009) and *CIPK8*, which has a regulatory role on *NRT2.1* (Hu et al., 2009). Glutamic acid (Glu) is important in N flow and also regulates root architecture by promoting root branching. This effect is antagonized by excess NO^−^_3_, and this antagonistic action is also regulated by *NRT1.1*-dependent signaling (Walch-Liu and Forde, 2008). Root development and branching is also conditioned to auxin signaling, and it appears that *NRT1.1* couples NO^−^_3_ sensing and auxin signaling.

There is recent evidence for other NO^−^_3_ sensors coordinating signaling events in *Arabidopsis*. *NRT2.1* displays *NRT1.1*-independent roles and is also unrelated to NO^−^_3_ uptake activity. This gene is a repressor of lateral root initiation under high sucrose and NO^−^_3_ supply, but surprisingly, this gene seems to sense abiotic stress and concomitantly suppresses biotic stress responses. We have demonstrated that the mutant *nrt2* is affected in bacterial effector manipulation (Camañes et al., 2012b). Mutation of *NRT2.1* has significant consequences in the transcriptome of *Arabidopsis* upon *P. syringae* infection, suggesting a complex regulatory function for the *NRT2.1* gene and also the NRT2.1 protein (Camañes et al., 2012b). Therefore, we hypothesize that *NRT2.1* should be designated a transceptor as previously proposed by Gojon et al. (2011). In *Arabidopsis*, the link between the immune system and nitrate transport is not fully understood. However, recent advances indicate two different relevant events. First, low nitrate treatment induces a rapid burst of ethylene production and upregulates *CTR1, EIN3*, and *EIL1* (Zheng et al., 2013). Second, we demonstrated that a low N treatment enhances *P. syringae* susceptibility (Camañes et al., 2012b), which may be related to the increase of ET-dependent signaling that is antagonistic to the SA-signaling that is recognized as an efficient defense against biotrophic pathogens (Pieterse et al., 2009).

Interactions with the plant immune system are not restricted to *NRT2.1*. Other NRT family members, such as *NRT2.6* show enhanced expression upon *Erwinia amylovora* infection (Dechorgnat et al., 2012). Despite high NO^−^_3_ levels that trigger *NRT2.6* expression, no nitrate-related phenotype has been associated with the *nrt2.6* mutant. Furthermore, *nrt2.6* is less tolerant to *E. amylovora* because of the reduced ability to produce H_2_O_2_ upon infection.

Some members of the *AMT1* family are high-affinity ammonium transporters. Of these, *AMT1.1* has the highest affinity for NH^+^_4_ (Shelden et al., 2001). The removal of N increases *AMT1.1* expression, and this correlates with an increase in NH^+^_4_ uptake. This function is affected by 30–40% in the insertional T-DNA mutant *amt1.1*. Interestingly, *amt1.1* plants are indistinguishable from wild type under optimal fertilization and growth conditions. Furthermore, *amt1.1* displays wild type levels of total N in the presence of N or after 4 days of N starvation (Kaiser et al., 2002). Although the ammonium supply may affect the plant responses to pathogens, to the best of our knowledge, no NH^+^_4_ transporter is involved in the plant immune system. *NRT2.1* and *AMT1.1* are the primary high-affinity transport systems (HATS) involved in NO^−^_3_ and NH^+^_4_ uptake, respectively (Cerezo et al., 2001; Kaiser et al., 2002), and reciprocal regulation between *AtNRT2.1* and *AtAMT1.1* expression has been observed (Camañes et al., 2012a). High-affinity ammonium and nitrate transporters act as sensors of low NH^+^_4_ and NO^−^_3_ in the root environment; therefore, thus the gene expression of *AMT1.1* and *NRT2.1* is enhanced upon low fertilization. Interestingly, its role in HATS under normal N supply conditions (>1 mM NO^−^_3_ or NH^+^_4_) is negligible (Gansel et al., 2001). Using an experimental system with optimal fertilization, we focused on the role of the high-affinity ammonium transporter gene in the *Arabidopsis* immune responses against the hemibiotrophic bacterium *P. syringae* and the necrotrophic fungus *P. cucumerina*. Metabolomic and genetic studies revealed an interplay between *AMT1.1* and plant resistance.

## Materials and methods

### Plant material and growth conditions

Seeds of the Arabidopsis accession Col-0 were obtained from the SALK institute (Alonso et al., 2003), the EMS *lin1* line (in Col-0 background) was generously supplied by Jocelyn Malamy (University of Chicago, EEUU, Little et al., 2005) and the line *nrt3.1* (salk_043672) in the Col-0 background was obtained from SALK institute (Alonso et al., 2003). The background *Col3-gl1* and the mutant *amt1.1* (in *Col3-gl1* background) were provided by Alain Gojon (INRA, Montpelier, France).

For the bioassays of bacterial resistance, 1 week after germination, the seedlings were transferred to 33-ml soil pots. The plants were cultivated at 20°C day/18°C night with 8.5 h of light (105 μE m^−2^ s^−1^) per 24 h and 60% relative humidity.

All plant genotypes were germinated in soil, and, 1 week after germination, seedlings were individually transferred to 33 ml pots containing commercial potting soil (TKS1, Floragard GmbH; http://www.floragard.de). Plants were cultivated at 20°C day/18°C night temperatures with 8.5 h of light (105 μE m^−2^ s^−1^) per 24 h and 60% relative humidity.

For fungal resistance assays. About 50 seeds were germinated in 33-ml soil pots. Plants were grown in the same conditions as described for bacterial experiments.

### Pathogen strains and resistance assays

The bacterial strain *Pseudomonas syringae* pv. *tomato* DC3000 was grown overnight at room temperature in King's B solid medium with appropriate antibiotics and diluted to the desired concentration with 10 mM MgSO_4_ for plant inoculation. The bacteria were used to infect 5-week-old (or otherwise mentioned) *Arabidopsis* plants by dipping in a suspension of 2.5 × 10^5^ colony-forming units (cfu)/ml using 0.02% Silwet L-77 (Tornero and Dangl, 2001), control plants were treated with Silwet accordingly. Three days after challenge inoculation, the disease level was determined by harvesting the plants a plating a range of dilutions in agar-KB medium containing rifampicin. After incubation at 28°C for 3 days, the number of rifampicin-resistant colony forming units per gram of infected leaf tissue was determined, and bacterial proliferation over the 3-day time interval was calculated.

The adapted strain of *P. cucumerina* BMM was kindly provided by Brigitte Mauch-Mani (Universitè de Neuchâtel, Switzerland). Two week old plants were sprayed with 1 × 10^3^ spores/mL and maintained at 100% relative humidity. Two days after inoculation leaves were sampled by freezing in liquid N_2_ and stored at −80°C until analysis. Disease rate was determined by microscopical analysis after tripan blue stainings of infected leaves (Flors et al., 2008). Leaves were classified in a disease ranking as explained in the figure legend.

### Growth conditions for starvation experiments

*Arabidopsis thaliana* was grown hydroponically as described in Lejay et al. (1999). The seeds were germinated directly on top of modified Eppendorf tubes filled with pre-wet sand. Plants were grown until the age of 5 weeks on a 1 mM NH_4_NO_3_ nutrient solution (repressed plants), which prevented any growth difference between the two genotypes (Lejay et al., 1999). Before inoculation experiments, plants were transferred for 48 h to N-free solution (de-repressed plants).

### HPLC-ESI full scan mass spectrometry (Q-TOF instrument)

Metabolome analysis was performed using an Acquity UPLC system (Waters, Mildford, MA, USA) interfaced to hybrid quadrupole time-of-flight (QTOF Premier). The LC separation was performed by HPLC SunFire C18 analytical column, 5 μm particle size, 2.1 × 100 mm (Waters). Analytes were eluted with a gradient of methanol and water containing 0.01% HCOOH. Chromatographic conditions and QTOF MS parameters were followed as described in Gamir et al. (2014).

### HPLC-ESI tandem mass spectrometry (triple quadrupole instrument). hormonal analysis

An Acquity ultra-performance liquid chromatography system (UPLC) (Waters, Mildford, MA, USA) was interfaced to a triple quadrupole mass spectrometer (TQD, Waters, Manchester, UK). The chromatographic separation conditions were closely related to those described previously. Chromatograpic conditions and TQD parameters were followed as described in Flors et al. (2008) and Gamir et al. (2012). Masslynx v 4.1 (Waters, Manchester, UK) software was used to process the quantitative data obtained from calibration standards and samples.

### Full scan data analysis

Raw data obtained from Masslynx software was transformed to.CDF using Databrigde provide by Masslynx package. The.CDF data was process with R for statistical computing using XCMS package for relative quantification (Smith et al., 2006). Principal Component Analysis (PCA) were used as explained in http://www.numericaldynamics.com/ as a tool to define major changes in the metabolome of the plant under priming condition during fungal infections.

For heatmap construction and clustering of metabolites it was used the software MarVis Filter and MarVis cluster (http://marvis.gobics.de/; Kaever et al., 2012).

### Kinetics of ^15^NH^+^_4_ influx

The kinetics of ^15^NH^+^_4_ influx as a function of external ^15^NH^+^_4_ concentrations ([^15^NH^+^_4_]_0_) was measured with [^15^NH^+^_4_]_0_ ranging from 0.02 to 0.8 mM. To kinetics studies control plants and de-repressed plants of three genotypes (Col-0 and *nrt3.1*) were used. Influx of ^15^NH^+^_4_ into the roots were assayed as described by Gazzarrini et al. (1999). Col-0 and *nrt3.1* plants with a normal fertilization or N depletion were sequentially transferred to 0.1 mM CaSO_4_ for 1 min and to complete nutrient solution (pH 6.0) containing ^15^NH^+^_4_ (98% atom excess ^15^N) for 5 min, at the concentrations indicated in figures. At the end of the ^15^N labeling, roots were washed for 1 min in 0.1 mM CaSO_4_ and were separated from shoots. The roots were dried at 70°C for 48 h, weighed, crushed in a hammer-mill and analyzed for total ^15^N content using an integrated system for continuous flow isotope ratio mass spectrometry (Euro-EA elemental analyser, EuroVector S.P.A.; and Isoprime mass spectrometer; GV Instruments). Root influx is expressed in μmol ^15^NH^+^_4_ (g root DW)^−1^ h^−1^. To kinetics studies data-transformation method based on the Michaelis-Menten formalism was used. The experiment was repeated three times.

### RNA extraction and real-time PCR analysis

Gene expression by quantitative real-time RT-PCR was performed using RNA samples extracted from root tissue using the RNA kit (Omega Bio-Tek Inc, Doraville, GA, USA) according to the manufacturer instructions. To avoid contaminating DNA, the samples were treated with DNAse I. A total of 1 μg of total RNA was annealed to oligo-dT and reverse transcribed using Omniscript Reverse Transcription kit (QIAGEN) to obtain cDNA. The sequences of the gene-specific oligonucleotides designed and used for real-time PCR are the following: *AMT1.1* forward: 5′acactgtggccagttaggcg3′ and reverse: 5′ccgtggggatgtctttgaga3′, *Tubuline (TUB)* forward: 5′cgattccgttctcgatgttgt3′ and reverse: 5′aatgagtgacacacttggaatcctt3′ and EF1α forward: 5′gtcgattctggaaagtcgacc3′ and reverse: 5′aatgtcaatggtgataccacgc3′. Real-time PCR was conducted using the QuantiTect™ SYBR Green PCR Kit (QIAGEN) and the SmartCycler II instrument (Cepheid). The experiment was repeated three times.

## Results

### Alteration in nitrate and ammonium transporters enhances basal resistance

**Figure 1:**
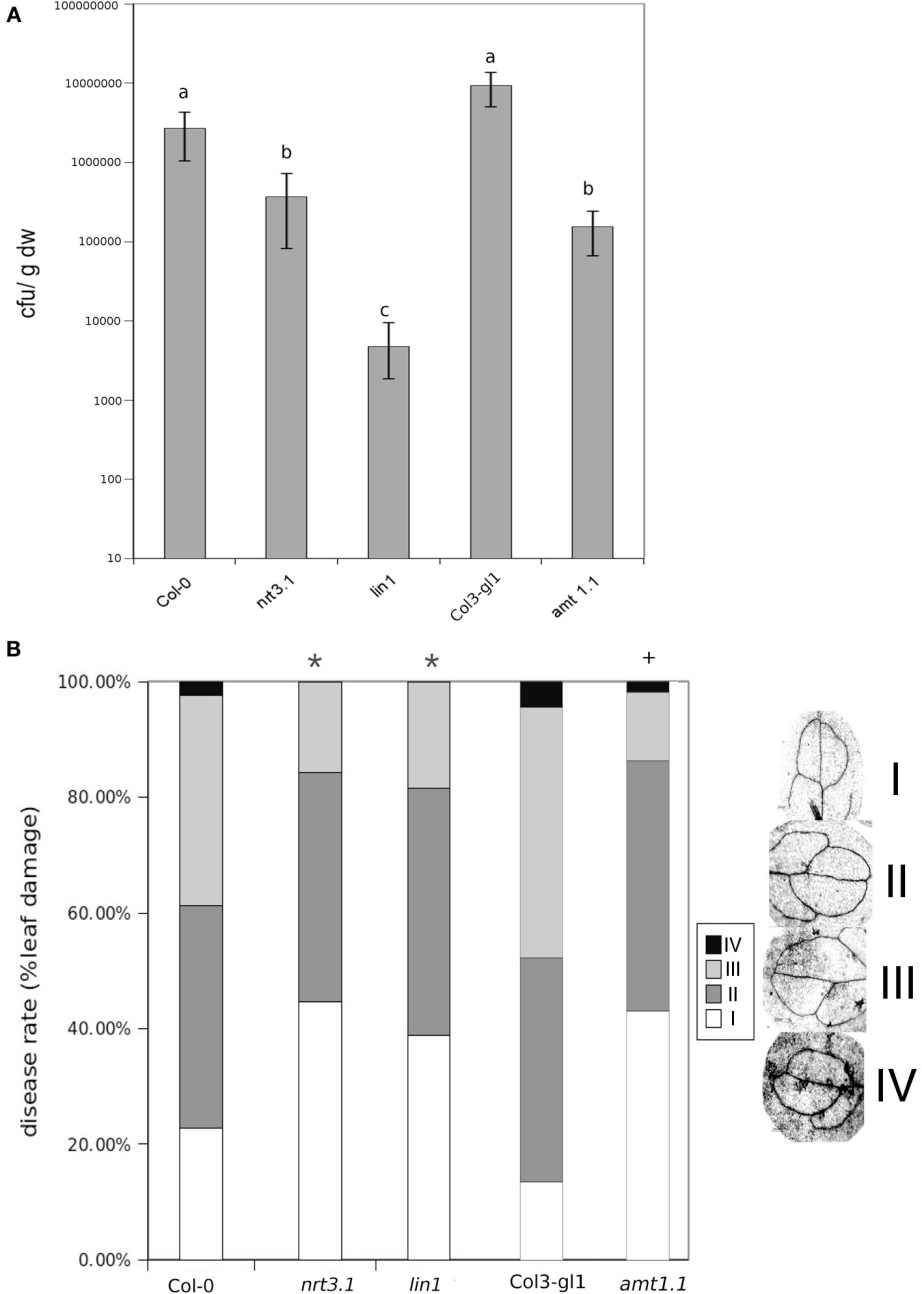
**Bacterial proliferation and disease rate in Col-0, *lin1, nrt3.1*, and *Col3-gl/1* and *amt1.1* plants infected with *P. syringae* and *P. cucumerina* respectivelly. (A)** Five week old plants were challenge-inoculated by dipping in a bacterial suspension of *P. syringae* at 2 × 10^5^ c.f.u. mL^−1^. The values presented are means (±SD) of the log of the proliferation values. Data represent the average of three independent experiments (*n* = 3). Different letters mean significant statistical differences (ANOVA, LSD test; *p* < 0.05). **(B)** Two week old plants were inoculated by spraying with 1 × 10^3^ spores × mL^−1^ with PcBMM. Disease symptoms were recorded by trypan-blue staining at 5 days post inoculation. Disease rate was ranked according to the infected leaf surface: level I no infection, level II less than 25% of infected leaf surface, level III between 25 and 50% of infected leaf surface, level IV more than 50% of infected leaf surface. The figure shows a representative experiment that was repeated three times with the same results. Data presented are the means of the percentage of diseased leaves per plant. Asterisk indicate statistically significant differences compared with non-induced control plants (*t*-test; ^*^p < 0.05; ^+^*p* < 0.05 with their respective controls; n~50 leaves).

**Figure 2:**
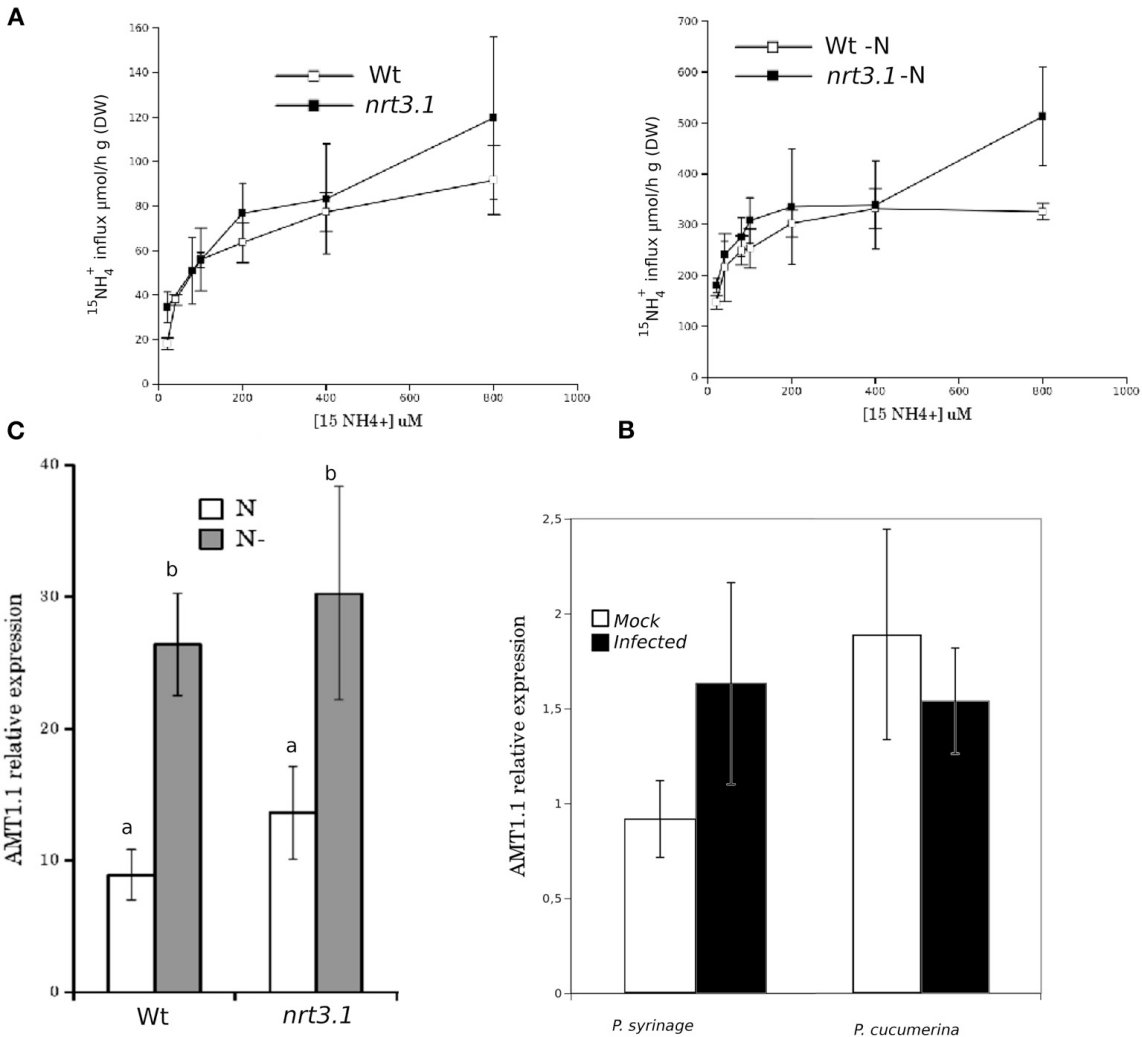
**(A)** Kinetics of ^15^NH^+^_4_ influx in Col-0 and *nrt3.1* roots in the low ^15^NH^+^_4_ concentration range. Plants were grown hydroponically with N supplied as 1 mM NH_4_NO_3_ during 6 weeks. After that one group of plants were transferred to 1 mM NH_4_NO_3_, other group of plants were transferred to N-free nutrient solution (-N) during 48 h. ^15^NH^+^_4_ influx was measured at different concentrations of external ^15^NH^+^_4_. Each data is the mean of 30 replicates ±SE. **(B)** Real-time PCR analysis of the expression of *AMT1.1* in Col-0 and *nrt3.1* plants fertilized normally along 5 weeks and exposed to N starvation 2 days (-N). The *AMT1.1* transcript levels were normalized to the expression of *TUB* measured in the same samples. The experiment was repeated using *EF1α* with similar results. Each bar represents average data with standard error bars from two technical replicates three independent experiments (*n* = 6). **(C)** Real-time PCR analysis of the expression of *AMT1.1* in mock and *P. syringae* or *P. cucumerina* wild type infected plants. The *AMT1.1* transcript levels were normalized to the expression of *TUB* measured in the same samples. The experiment was repeated using *EF1α* with similar results. Each bar represents average data with standard error bars from two technical replicates three independent experiments (*n* = 6).

It has been reported that deletions in *NRT2.1* and *NRT2.2* cause the reduced susceptibility of *Arabidopsis* to *P. syringae* (Camañes et al., 2012b). *NRT2.1* has been proposed as a transceptor that delivers and coordinates distal signaling that affects primary metabolism and defense signaling (Gojon et al., 2011; Camañes et al., 2012b). The function of *NRT2.1* in transporting nitrate is also influenced by the membrane protein *NRT3.1*, which is not a transporter itself, but its mutation significantly affects the HATS of nitrate (Okamoto et al., 2006; Orsel et al., 2006). To determine whether deficiencies in *NRT3.1* and the ammonium transporter gene *AMT1.1* have consequences in the basal resistance, we characterized the responses of the mutant lines *nrt3.1* and *amt1.1* toward *P. syringae* and *P. cucumerina*. The enhanced resistance mutant *lin1* (blocked in *NRT2.1*) was used as a control. All three mutants showed enhanced resistance against both *P. syringae* and *P. cucumerina* compared to their respective control wild type plants (Figures 1A,B). The mutant *lin1* was more resistant to the bacterium, whereas *nrt3.1* and *amt1.1* showed significantly reduced susceptibility, although lower than *lin1*. Because *NRT3.1* influences nitrate transport, we tested whether it also affects ammonium transport. The mutant *nrt3.1* showed wild type ^15^NH^+^_4_ uptake kinetics both with N and after 48 h of N depletion (Figure 2A). In addition, we also determined whether the *AMT1.1* gene is upregulated in the absence of N under the same experimental conditions. N depletion induces (de-represses) *AMT1.1* expression in wild type plants (Gazzarrini et al., 1999), whereas *nrt3.1* shows wild type *AMT1.1* expression (Figure 2B). Both results suggest that *NRT3.1* does not modulate ammonium kinetics uptake and that it does not alters *AMT1.1* de-repression.

Finally, we tested whether infection with *P. syringae* or *P. cucumerina* affects *AMT1.1* expression in a manner that may act as targets for bacterial or fungal effectors. Following infection, *AMT1.1* expression was not significantly modified by any of the pathogens (Figure 2C).

### Nitrogen starvation enhances *P. syringae* susceptibility but does not affect *P. cucumerina* resistance

**Figure 3:**
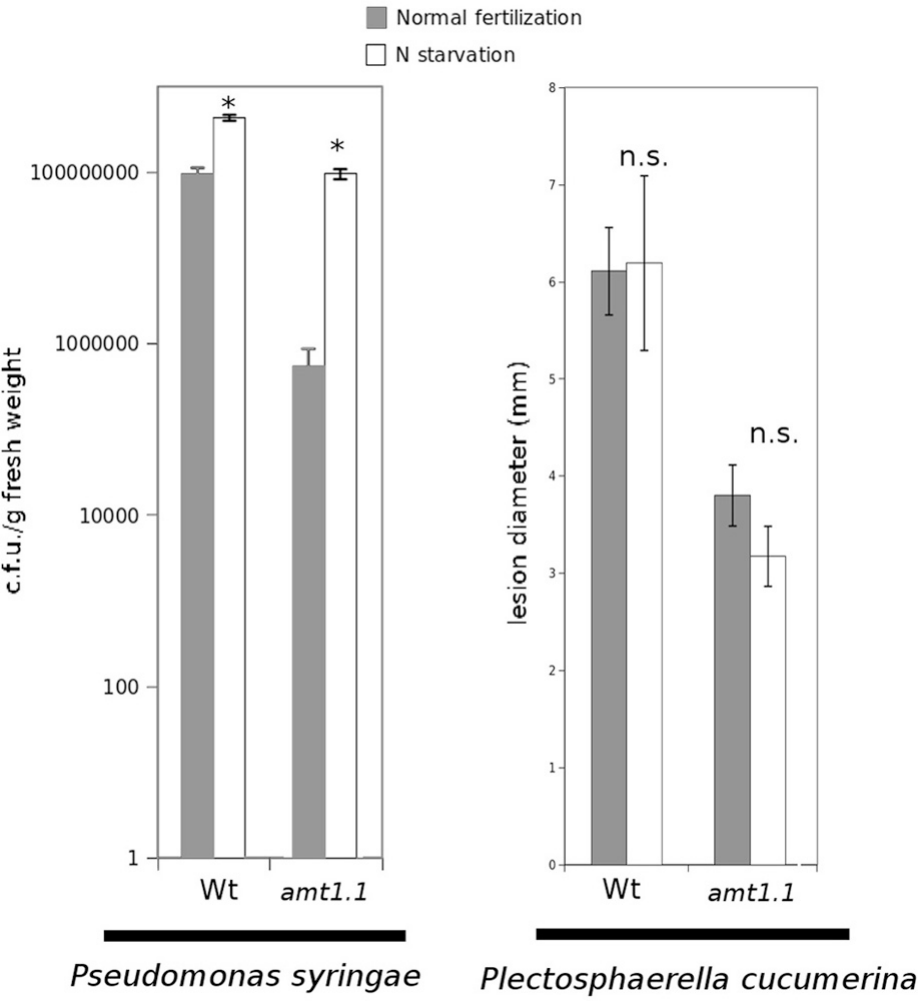
***P. syringae* proliferation and lesion diameter cause by *P. cucumerina* in *Col3-gl1* and *amt1.1* plants fertilized normally (Normal fertilization) along 5 weeks and exposed to N starvation 2 days before inoculation (N starvation)**. Hydroponically growing plants were challenge-inoculated either with a bacterial suspension of *P. syringae* at 2.5 × 10^5^ c.f.u./ml or with a drop of 1 × 10^5^ spores × mL^−1^ of PcBMM. Data represent the average of three independent experiments (*n* = 3). The values presented are means of infected plants (±SD). Asterisk indicates statistically significant differences (LSD test; *p* < 0.05).

Previously, we determined that nitrate starvation for 48 h increased the susceptibility of *Arabidopsis* toward *P. syringae* and that *nrt2.1* was also impaired (Camañes et al., 2012b). To determine whether N depletion affects the basal resistance against both pathogens and to determine the contribution of the ammonium transporter *AMT1.1* to this interaction, we performed a hydroponic experiment by inducing N depletion for 48 h. According to our previous study (Camañes et al., 2012b), wild type starved plants were more susceptible to *P. syringae* (Figure 3), whereas they were not affected in their resistance to the fungus (Figure 3). Notably, *amt1.1* starved plants also showed increased susceptibility against *P. syringae*, whereas their resistance to the fungus was not affected.

### *amt1.1* displays altered hormonal defense responses against *P. syringae* and *P. cucumerina*

**Figure 4:**
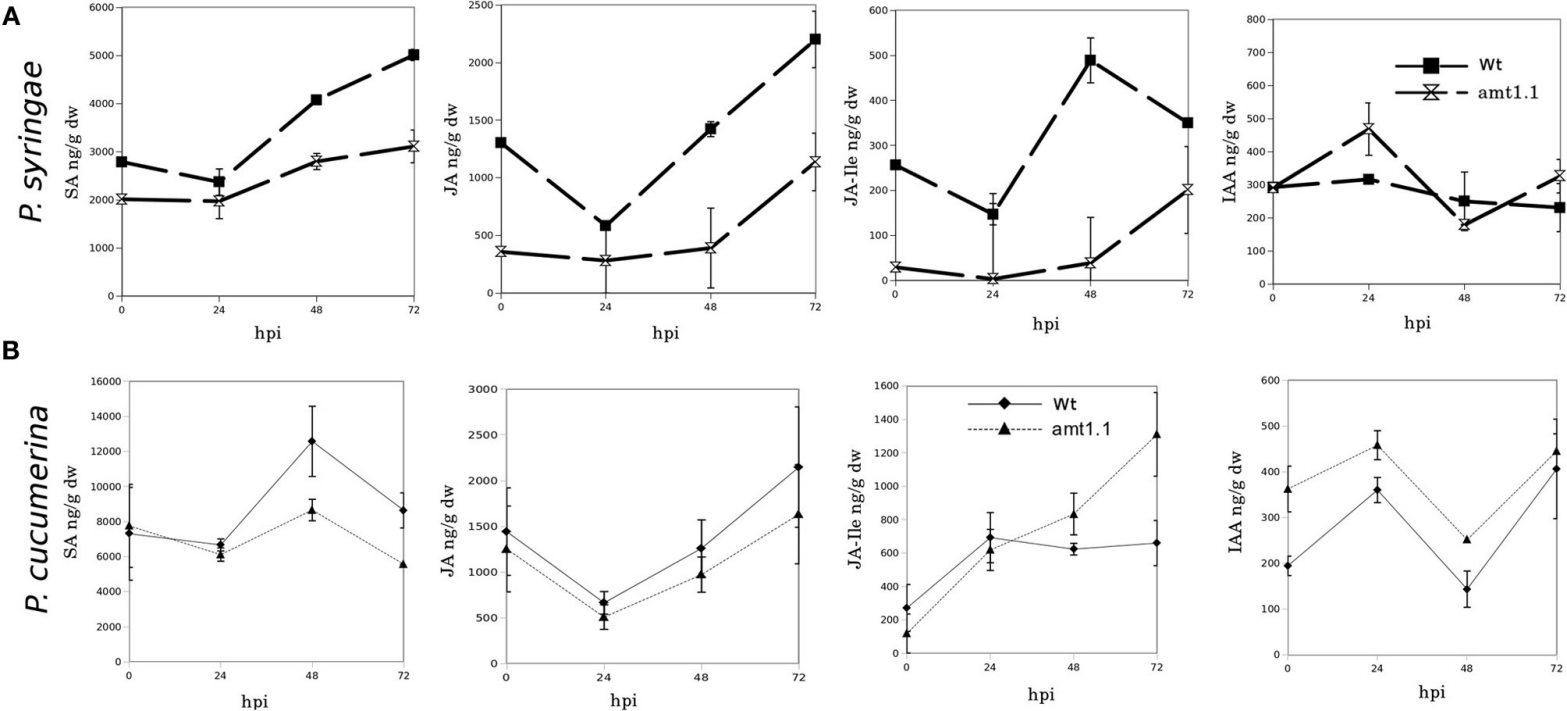
**SA, JA, JA-Ile, and IAA profiling upon *P. syringae* and *P. cucumerina* infection**. Plants were challenged as described in Figure 1. Both mock and pathogen infected plants were harvested at different time-points. Freeze dried material was processed for a targeted quantification analysis by TQD-MS. The concentration of the hormones was determined in all the samples by normalizing the chromatographic area for each compound with the dry weight of the corresponding sample. Leaf material from 15 individual plants for *P. syringae* **(A)** resistance assays and 150 plants for *P. cucumerina* **(B)** resistance assays were pooled together for each treatment × genotype combination. Data represent average three independent experiments ±SD; *n* = 3.

**Figure 5:**
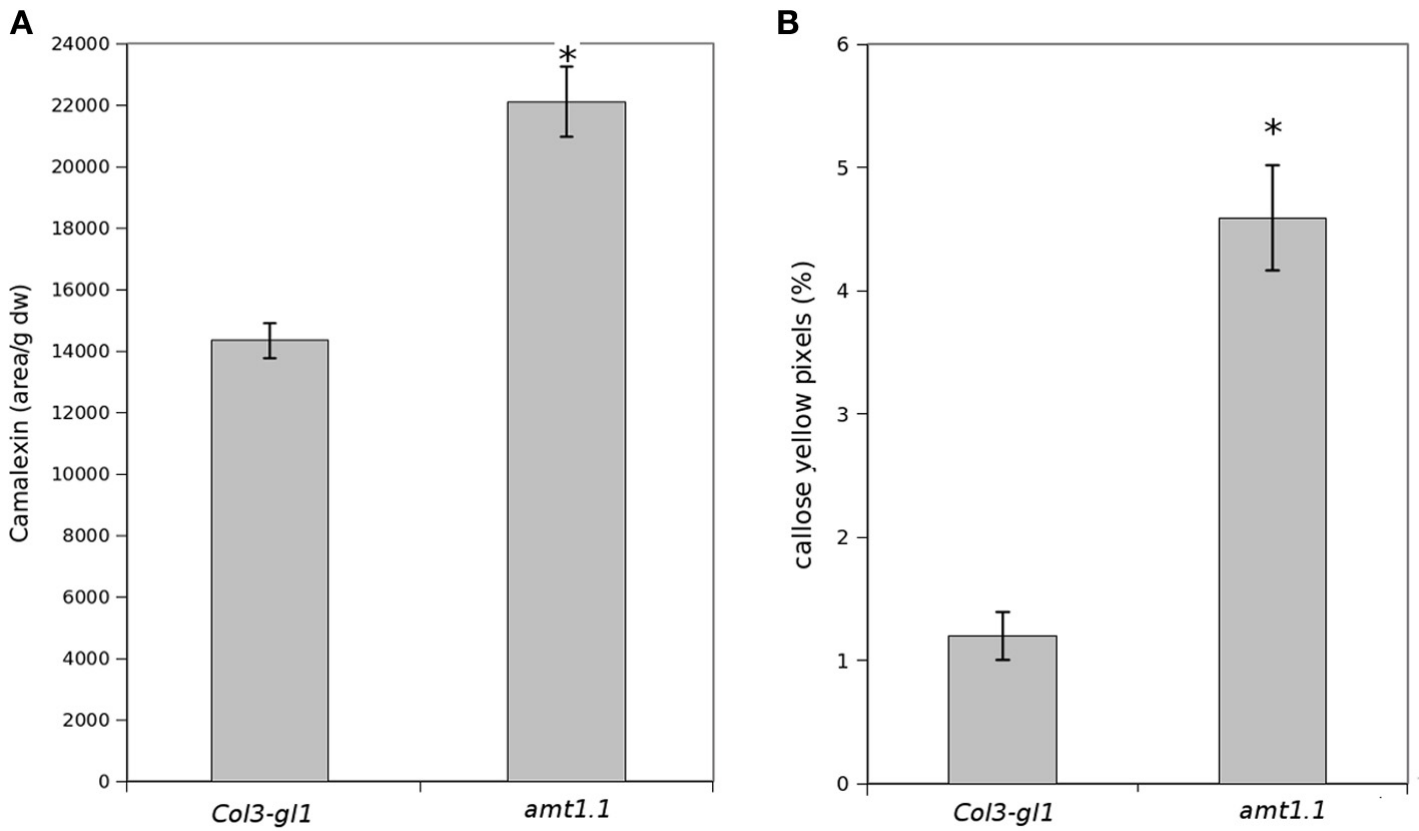
**Camalexin and callose levels upon *P. syringae* and *P. cucumerina* infection**. Plants were challenged as described in Figure 1. Either mock or pathogen infected plants were harvested at 48 hpi. **(A)** Freeze dried material was processed for a targeted quantification analysis of camalexin by TQD-MS. The relative concentration was determined in all the samples by normalizing the chromatographic area for each compound with the dry weight of the corresponding sample. Leaf material from 150 individual plants were pooled together for each treatment × genotype combination. Data represent average three independent experiments. **(B)** Callose was visualized by aniline blue staining and epifluorescence microscopy (UV). Quantification was performed by determining the number of yellow pixels per million pixels corresponding to pathogen-induced callose on digital photographs of infected leaf areas. Asterisk indicates statistically significant differences (LSD test; *p* < 0.05). Data shown are means (±SD; *n* = 20) of the relative number of yellow pixels per photograph.

Because *amt1.1* showed reduced susceptibility and enhanced resistance toward the bacterium and the fungus, respectively, compared to the wild type plants, we analyzed the main hormones involved in defense signaling. Surprisingly, the SA, JA, and JA-Ile levels remained lower in the *amt1.1* plants infected with *P. syringae* for the entire experiment compared to the wild type plants (Figure 4A). Indoleacetic acid (IAA), however, did not significantly change in the *amt1.1* plants. A reduction of JA and JA-Ile in *amt1.1* plants suggests that this mutation may counteract bacterial effectors by enhancing resistance. However, this hypothesis is unlikely because the SA levels remained lower in the mutant. Therefore, the hormonal analysis did not clearly explain the reduced susceptibility of *amt1.1* to the bacterium. By contrast, the hormonal responses of the mutant against the fungus may contribute to resistance. JA-Ile and IAA following infection are increased compared to the wild type plants; however, SA at 48 and 72 hpi is reduced in the mutant infected with *P. cucumerina* (Figure 4B). We also tested other common defense responses that effective against *P. cucumerina*, such as the camalexin levels and callose accumulation (Ton and Mauch-Mani, 2004; Gamir et al., 2014). Both defensive responses were strongly enhanced in the mutant response to the pathogen. Camalexin and callose in *amt1.1* remained significantly higher at 48 hpi (Figure 5).

### Metabolomic profiling of *amt1.1* in response to pathogen attack

The hormonal profile of *amt1.1* in response to bacterial infection did not explain its enhanced resistance; therefore, we performed full metabolomic profiling to understand the metabolic changes that may contribute to the resistance of this mutant. For *P. cucumerina*, the classical defenses against necrotrophs, such as JA-Ile, callose, and camalexin, over-accumulated in *amt1.1* plants infected with the fungus. We also performed metabolic profiling following infection with the fungus to determine the extent to which *amt1.1* affects the responses to fungal infection. We used untargeted reverse-phase liquid chromatography-mass spectrometry (HPLC-QTOF-MS) for the profiling. *Col3-gl1* and *amt1.1* plants were either mock or *P. syringae* or *P. cucumerina* infected. Metabolomic analysis and data reporting were performed as described by Pitzschke and Hirt (2010), Fernie et al. (2011), and Kaever et al. (2012).

**Figure 6:**
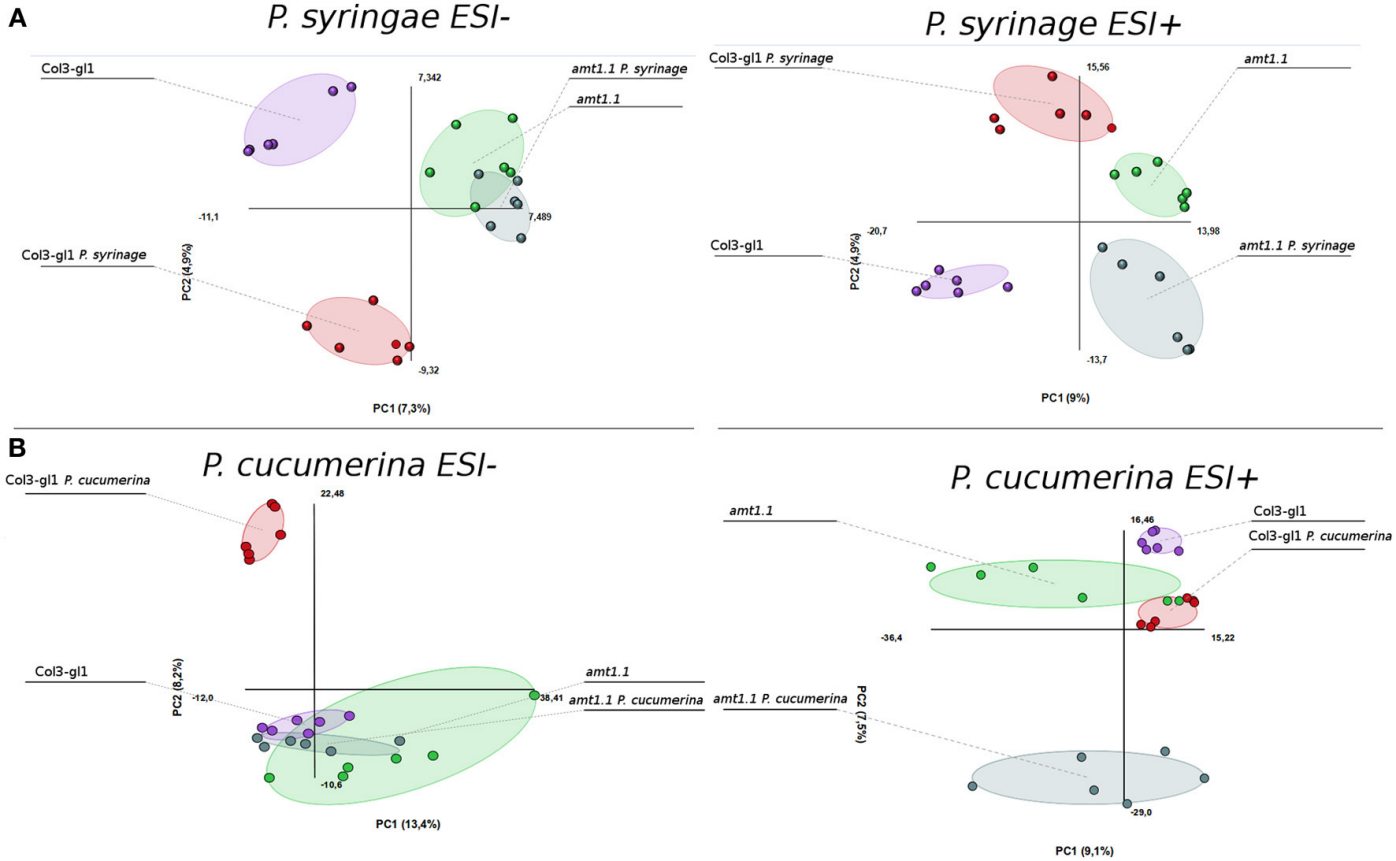
**Non-supervised Principal Component Analysis**. (PCA) analysis representation of major sources of variability of ESI+ and ESI− signals obtained from a non-targeted analysis by HPLC-QTOFMS to monitor metabolomic changes during bacterial **(A)** and fungal invasion **(B)**. **(A)** Five week old plants were dip inoculated with *P. syringae* with 2.5 × 10E^5^ c.f.u/ml. 48 hpi Leaf material from 15 individual plants were pooled together for each treatment × genotype combination. **(B)** Two week old plants were sprayed inoculated with 10E^3^ spores/ml of *P. cucumerina* and samples for analysis were collected 48 hpi. Leaf material from 150 individual plants were pooled together for each treatment × genotype combination. Data points represent two technical replicates from three independent experiments (biological replicates; *n* = 6) injected randomly into the HPLC-QTOFMS. The signals corresponding to different treatments were compared using the non-parametric Kruskal-Wallis test, and only data with a *P*-value lower than 0.01 between groups was used for subsequent processing.

The acquired raw data were transformed into.cdf files using Databridge from the Masslynx 4.1 software (Masslynx 4.1, Waters). These data were subsequently subjected to analysis using the free software R for statistical purposes. The signals from the positive and negative electro-spray analysis (ESI+; ESI−) were processed separately, these ionization modes assure that all compounds that tend to form either cations or anions will be detected by the mass spectrometer. We performed a PCA of all signals obtained in ESI+ and ESI−. The infection with *P. syringae* and *P. cucumerina* induced strong changes in the metabolome of the wild type plants and *amt1.1* plants that differed for the positively and negatively ionized compounds (Figure 6). The impact of the infection in ESI+ appeared stronger in *amt1.1* upon *P. cucumerina* infection compared with *P. syringae*; however the changes in ESI− are more subtle because there is an overlap in the variability of *amt1.1* with or without infection independently of the pathogen.

Notably, in the absence of infection, the basal metabolomes of *Col3-gl1* and *amt1.1* do not overlap in any of the PCA with the exception of ESI− in the seedlings. This suggests that many metabolites are already altered in *amt1.1* in the absence of infection, and these changes may contribute to its enhanced resistance, discarding a general priming phenomenon to explain the phenotypes observed against both pathogens. Additionally, the wild type plants and *amt1.1* plants show differences in the basal responses between both experiments without inoculation, which may be due to a difference in the age of the plants (5 and 2 week-old plants for the *P. syringae* and *P. cucumerina* experiments, respectively) but also to the different mock treatments (dip inoculated Silwet for *P. syringae* and sprayed 10 mM MgSO_4_ for *P. cucumerina*). A heatmap analysis was performed to identify metabolites involved in the resistance phenotypes of *amt1.1*, and the clusters of metabolites that over-accumulated in *amt1.1* either in the absence or presence of the infection were selected (Figure S1). Clusters from the heatmaps that showed overaccumulated compounds in the mutant (red color) were selected for a detailed metabolic study and pathway analysis.

Prior to identifying the signals in the selected clusters, we performed a detailed analysis of the amino acids in the metabolome. For such purpose, we constructed a library of amino acid standards for the identity assignation using the exact mass provided by the Q-TOF analyser and the retention time parameter (Gamir et al., 2014).

**Figure 7:**
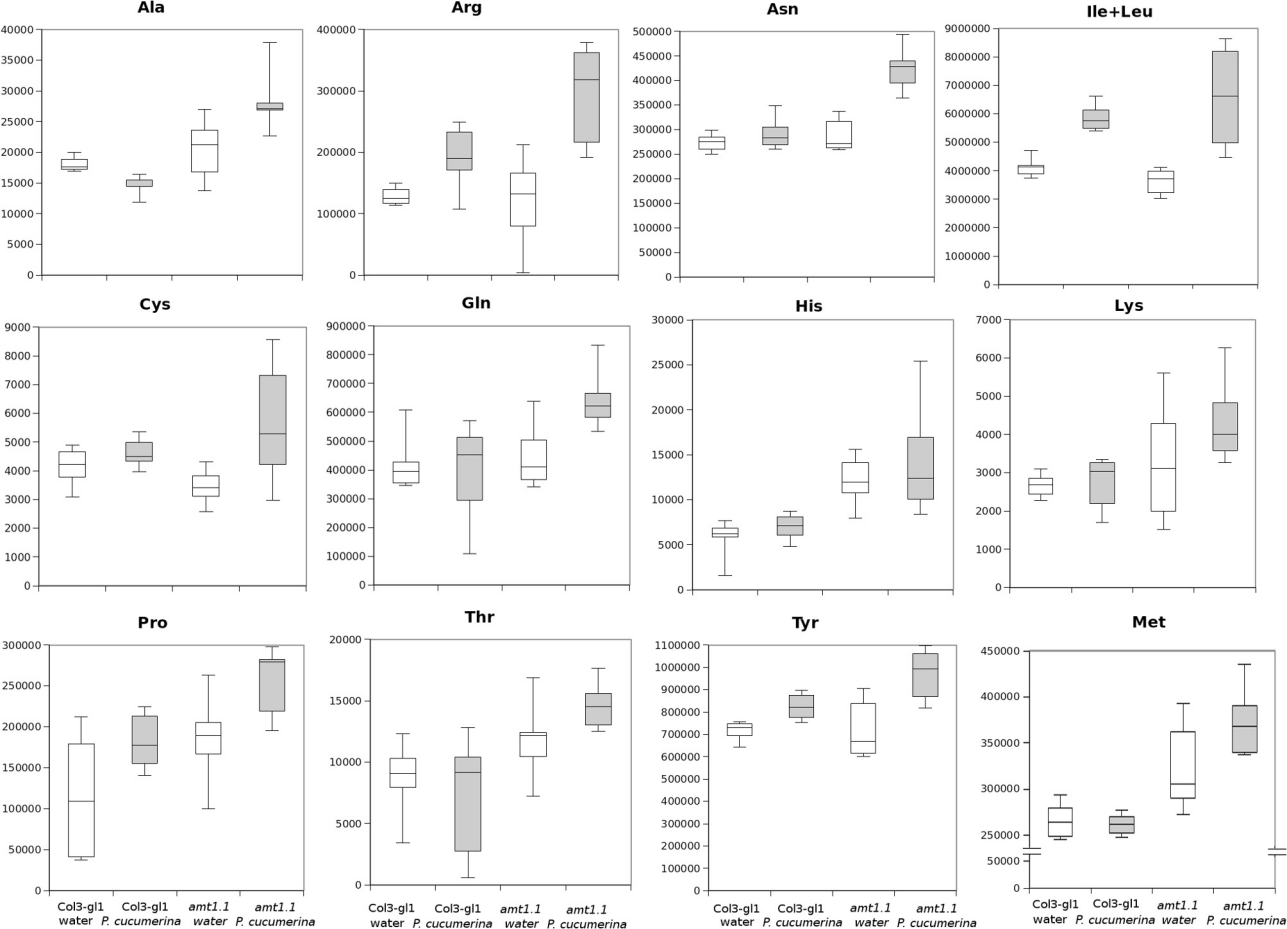
**Aminoacid profiling upon *P. cucumerina* infection**. Two week old *Col3-gl/1* and *amt1.1* plants either mock or *P. cucumerina* inoculated were processed for relative quantification analysis by HPLC-QTOFMS data. The concentration of the metabolites was determined in all the samples by normalizing the chromatographic area for each compound with the dry weight of the corresponding sample. White bars are mock inoculated and filled bars are *P. cucumerina* infected plants. Leaf material from 150 individual plants were pooled together for each treatment × genotype combination. Boxplots represent average three independent experiments with two technical replicates (*n* = 6).

**Figure 8:**
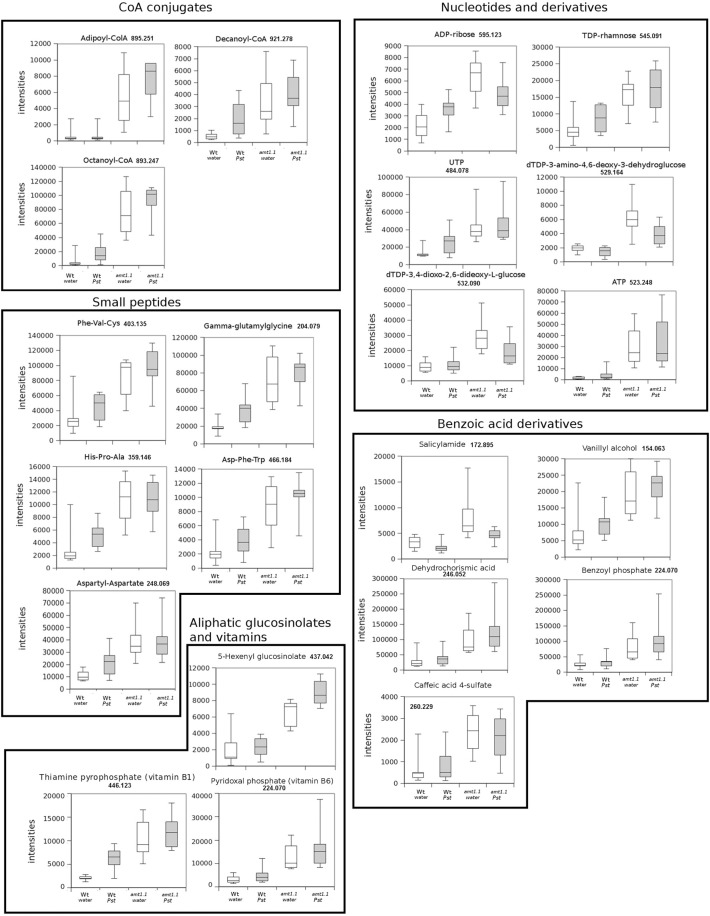
**Profiling of the main hits in the overaccumulated compounds upon *P. syringae (Pst)* infection in *amt1.1* compared with *Col3-gl1***. Five week old *Col3-gl1* and *amt1.1* were infected as described in Figure 1. After 48 hpi plants were processed for relative quantification analysis by HPLC-QTOFMS data. The concentration of the metabolites was determined in all the samples by normalizing the chromatographic area for each compound with the dry weight of the corresponding sample. The compounds were tentatively identified using the exact mass criteria using the METLIN and Massbank databases. The compounds were grouped by metabolic pathways according to KEGG and AraCyc databases. White bars are mock inoculated and filled bars are *P. syringae* infected plants. Leaf material from 15 individual plants were pooled together for each treatment × genotype combination. Boxplots represent average three independent experiments with two technical replicates. Only data showing a *p*-value below 0.05 after a Kuskal Wallys test were used for pathway assignation (*n* = 6).

**Figure 9:**
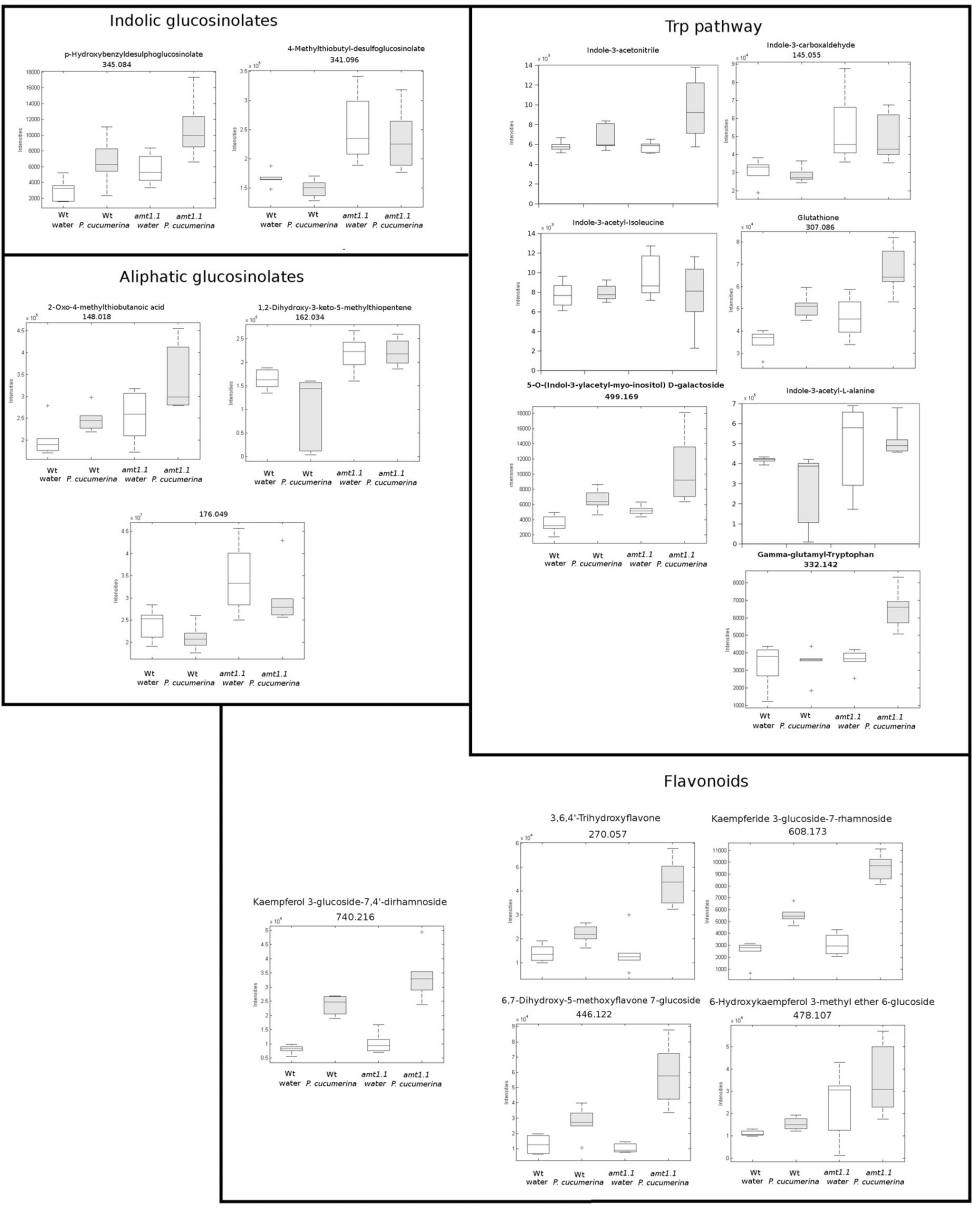
**Profile of the main hits in the overaccumulated compounds upon *P. cucumerina* infection in *amt1.1* compared with *Col3-gl1***. Two week old *Col3-gl1* and *amt1.1* were infected as described in Figure 1. After 48 hpi plants were processed for relative quantification analysis by HPLC-QTOFMS data. The concentration of the metabolites was determined in all the samples by normalizing the chromatographic area for each compound with the dry weight of the corresponding sample. The compounds were tentatively identified using the exact mass criteria using the METLIN and Massbank databases. Those compounds tentatively identified have the exact mass indicated. Te compounds fully identified have no reference to the mass. The compounds were grouped by metabolic pathways according to KEGG and AraCyc databases. White bars are mock inoculated and filled bars are *P. cucmerina* infected plants. Leaf material from 150 individual plants were pooled together for each treatment × genotype combination. Boxplots represent average three independent experiments with two technical replicates. + indicates outliers. Only data showing a *p*-value below 0.01 after a Kuskal wallys test were used for pathway assignation (*n* = 6).

In response to *P. syringae*, all the amino acids, together with azelaic acid and pipecolic, were not increased in the *amt1.1* mutant in response to the bacterium (data not shown). However, a different profile was observed following *P. cucumerina* infection. The majority of amino acids were increased in *amt1.1* plants with or without infection (Figure 7). Pro, Thr, His, and Met were previously elevated in the absence of infection and this increase occurred until 48 hpi. Ala, Arg, Asn, Ile+Leu (not separated in our chromatographic analysis), Cys, Gln, Lys, and Tyr showed a primed profile because they remained at the same levels in the wild-type plants before the inoculation; however, after infection, *amt1.1* displayed elevated levels compared to *Col3-gl1*. Only Asp, Glu, Phe, Trp, and Val in the mutant remained at the same levels as wild type infected plants (data not shown). Next, we performed a full comparative analysis of the metabolome. To identify the compounds, we used a library of standards with both the exact mass provided by the Q-TOF detector and the retention time. For the compounds with no available standards, we used the exact mass and fragmentation spectrum, when available, from the Massbank and Metlin databases. For such compounds, we assigned a tentative identification. After the signals were either exactly or tentatively identified, the compounds were searched against the Aracyc (http://pathway.gramene.org/gramene/aracyc.shtml) and Kegg (http://www.genome.jp/kegg/) databases to assign a putative metabolic pathway and a biological function. The analysis of the hormones or amino acids in *amt1.1* yielded a plausible explanation for its reduced susceptibility to *P. syringae*. However, the selection of the metabolite clusters from the heatmap that over-accumulated in *amt1.1* compared to the wild type plants following infection provides valuable information. Following the tentative identification of metabolites and their classification into metabolic pathways of *Arabidopsis*, we observed that fatty acid-CoA conjugates were increased in *amt1.1* either in the absence or presence of *P. syringae* (Figure 8). Nucleotides, such as ATP or UTP were also increased in *amt1.1*. Interestingly, three chemical species derived from TDP were also found for the *amt1.1* over-accumulated compounds. Dipeptides and tripeptides were another group of interest, which was surprising because the free amino acids in *amt1.1* are not different from the wild type plants. Although SA was not increased in the mutant, several benzoyl compounds over-accumulated in *amt1.1*, both with and without infection. The metabolic pathways with a higher number of hits in the cluster of over-accumulated compounds in *amt1.1* were the flavonoid and phenylpropanoid phytoalexins (Table 1). Although infection induced a strong increase in these compounds in *Col3-gl1, amt1.1* showed higher levels in the absence of infection. Finally, an aliphatic glucosinolate and vitamins B1 and B6, all of which are involved in defense, were also increased in the mutant (Figure 8). For the interaction with *P. cucumerina*, the identified compounds matched four secondary metabolism pathways of *Arabidopsi*s, indolic glucosinolates, aliphatic glucosinolates, the Trp pathway and phenylpropanoids (with a total of 19 compounds) (Figure 9). Notably, many secondary metabolites in these groups accumulated in *amt1.1* without infection, and all were then either increased or maintained in response to *P. cucumerina* in *amt1.1* plants compared to wild type plants. In addition to these pathways, although there were fewer hits, tentative identification of the metabolites showed that *amt1.1* responded to infection by increasing three mevalonic acid derivatives, four nucleotide intermediaries, three shikimic acid metabolites, two polyamines, and several hormones, such as GA3, GA-A58, and ABA (Figure S2).

**Table 1:** **Phenylpropanoid profiling upon *P. syringae* infection**.

**Neutral mass**	***Col3-gl1*-Mock**	***Col3-gl1-P. syringae***	**amt1.1-Mock**	**amt1.1-*P. syringae***	**Tentative identification**
**ESI**−					
868.141	5717.574 ± 1339.009	7903.077 ± 1171.697	15069.108 ± 3900.508	10608.243 ± 1332.910	Quercetagetin 7-methyl ether 3-(2‴-caffeoylglucosyl)-(1→2)-glucuronide
784.183	1993.974 ± 416.230	5584.543 ± 1443.404	6600.123 ± 1439.933	5310.700 ± 908.973	6-Hydroxyluteolin 7-[6″-(3-hydroxy-3-methylglutaryl)glucoside]-3-glucuronide
920.272	1741.940 ± 548.621	4257.727 ± 1356.214	9965.213 ± 1679.580	8535.086 ± 1714.162	Quercetin 3-(2‴-caffeylsambubioside)-7-glucoside
546.072	2872.295 ± 527.433	5379.807 ± 1033.293	10884.914 ± 2827.895	20974.750 ± 3776.673	4,6-Dideoxy-4-oxo-dTDP-D-glucose
982.276	1986.003 ± 537.055	6063.620 ± 1171.669	11885.748 ± 1351.898	15605.830 ± 897.168	Malvidin-3-(p-coumaroyl)-rutinoside-5-glucoside
356.221	3751.581 ± 770.196	2451.929 ± 1357.613	11677.959 ± 1955.930	11193.410 ± 1678.297	4,8,11,14-Eicosatetraenoic acid, 6-hydroxy-, (E,Z,Z,Z)-
402.132	97604.298 ± 15455.088	190757.113 ± 33780.888	343773.018 ± 49529.885	404922.286 ± 53046.318	7-Hydroxyflavanone beta-D-glucopyranoside
892.242	16249.517 ± 9694.477	39546.429 ± 13717.218	164163.569 ± 34206.807	186117.251 ± 20320.571	Palargonidin 3-(6″-ferulylglucoside)-5-(6‴-malonylglucoside)
528.126	834.255 ± 209.720	1438.870 ± 373.901	6122.663 ± 1052.023	6546.308 ± 1472.074	4,2′-Dihydroxy-3,4′,6′-trimethoxychalcone 4-glucoside
344.123	6814.904 ± 2501.614	9237.129 ± 1791.413	17129.393 ± 2994.036	18527.512 ± 1827.166	5,6,7,4′-Tetramethoxyflavanone
460.129	25299.855 ± 13806.666	40022.020 ± 15639.629	158482.998 ± 18759.482	169688.892 ± 18730.315	7,8,3′,4′-Tetramethoxy-6″,6″-dimethylpyrano[2″,3″:5,6]flavone
894.252	2544.518 ± 1377.062	5227.236 ± 2004.211	25842.316 ± 5738.193	29016.835 ± 3447.355	Genistein 7,4′-bis(O-glucosylapioside)
622.151	2809.060 ± 709.473	7451.498 ± 1212.428	12048.485 ± 1529.533	12526.350 ± 1257.233	Kaempferol 3-[2′”-acetyl-alpha-L-arabinopyranosyl-(1→6)-galactoside]
**ESI+**					
292.042	1186.017 ± 392.336	1807.786 ± 476.655	4104.516 ± 661.600	4953.756 ± 1425.747	5,7,3′-Trihydroxyisoflavone
360.181	853.215 ± 287.879	1370.833 ± 540.537	6396.268 ± 2111.103	4320.185 ± 1595.289	5,2′,3′-Trihydroxy-3,7,8-trimethoxyflavone
306.072	5958.788 ± 1705.843	7086.008 ± 2216.577	23547.293 ± 5398.893	19686.483 ± 5614.565	Pelargonidin
400.116	30044.197 ± 4429.060	48455.063 ± 8051.467	89620.850 ± 16202.253	69018.310 ± 10018.920	Flavonol 3-O-D-galactoside
544.609	1029.246 ± 505.676	2338.742 ± 704.251	5440.121 ± 1619.737	5301.904 ± 2113.984	Quercetin 3-glucoside-3′-sulfate
498.147	65764.676 ± 21821.696	45436.512 ± 10350.166	202317.111 ± 40047.133	192242.002 ± 55136.009	Quercetin 7-methyl ether 3,3′-disulfate
460.138	12122.235 ± 2423.017	16230.111 ± 3295.099	27447.715 ± 4518.375	24785.374 ± 4133.228	Luteolin 4′-methyl ether 7,3′-disulfate
636.387	137818.486 ± 17769.746	86637.426 ± 7725.291	71514.777 ± 4284.190	63127.380 ± 9084.052	flavonoid Isoscutellarein 4′-methyl ether 8-(2″,4″-disulfatoglucuronide)

*Five week old plants either mock Col3-gl/1 and amt1.1 or P. syringae inoculated (Col3-gl/1 Pc and amt1.1 Pc) plants were processed for relative quantification analysis by HPLC-QTOFMS data. Tentative identification of signals was performed by contrasting the exact mass in the METLIN and Massbank databases. The concentration of selected signals within the phenylpropanoid metabolism was determined in all the samples by normalizing the chromatographic area for each compound with the dry weight of the corresponding sample. Data show average values of three independent biological replicates containing pools of 15 plants in each replicate are shown with their standard deviation*.

## Discussion

We previously reported (Camañes et al., 2012b) that under optimal nutritional conditions, the total contents of N in *nrt2.1* and *amt1.1* plants are identical, which indicates that the N content is not responsible for altering the basal resistance in the mutants. Based on our previous study, we performed all resistance experiments, except otherwise noted, under the optimal fertilization conditions. First, we demonstrated that mutations in several genes related to N transport resulted in an increased resistance against two pathogens with different lifestyles. The HATS of nitrate uses a membrane complex in which the NRT3.1 protein modulates the function of *NRT2.1*, and mutation of *NRT3.1* results in the most significant losses of HATS function compared to *NRT2.1* mutations (Orsel et al., 2006). Therefore, we tested two different hypotheses. First, we tested whether this gene may also regulate ammonium transport, and second, we tested whether *NRT3.1* is involved in the plant defense response. The uptake experiments with ^15^NH^+^_4_ showed that the absorption of ammonium in *nrt3.1* does not differ from Col-0 plants, which suggests that it is not involved in ammonium HATS. We also demonstrated that *AMT1.1* gene expression with or without N was similar for Col-0 and *nrt3.1* plants. These observations confirm that this gene is not involved in *AMT1.1* modulation. Because *nrt3.1* plants show resistance against *P. cucumerina* and *P. syringae*, this suggests that it may have overlapping mechanisms with *NRT2.1* to stimulate defense; however, this hypothesis must be confirmed.

We ascertained the influence of *AMT1.1* on plant resistance because our experiments suggest that *AMT1.1* acts as a transceptor to coordinate plant tolerance against N depletion and defense against pathogens as suggested previously for NRT1 and NRT2.1 (Gojon et al., 2011; Camañes et al., 2012b). The mutant *amt1.1* showed reduced susceptibility against *P. syringae*, and surprisingly, *amt1.1* showed strong resistance toward *P. cucumerina* infection compared to wild type plants. Previously, we demonstrated that environmental factors contribute to the enhanced expression of *NRT2.1*. For example, N depletion affects the resistance of *Arabidopsis* to *P. syringae*. Although the depletion of N induces *AMT1.1* gene expression (Figure 2), it also increased the susceptibility in *amt1.1* plants, suggesting that it is the absence and not the overexpression of *AMT1.1* that alters resistance mechanisms. Notably, neither infection with *P. syringae* or *P. cucumerina* significantly altered the *AMT1.1* transcripts. We tested whether this also applies to resistance against necrotrophs. The enhanced resistance of *amt1.1* against *P. cucumerina* was not altered by N depletion; therefore, the absence of these genes impacts *Arabidopsis* defense against this pathogen but not their overexpression, suggesting posttranscriptional or posttranslational regulation. This is not so surprising because NRT1.1 and NRT2.1 have a complex protein regulation mechanism mediated by proteins, such as CIPK23 and NAR2.1 (Okamoto et al., 2006; Ho et al., 2009).

To identify the molecular mechanisms for the *amt1.1* phenotypes, we performed several targeted and non-targeted metabolomic studies. For the targeted studies, hormones regulate the plant immune system (Pieterse et al., 2009). Active SA-dependent responses against *P. syringae* promote resistance in *Arabidopsis*. Additionally, because of the existing negative crosstalk between the SA and JA signaling pathways, resistance against biotrophs is associated with the concomitant downregulation of JA and JA-Ile levels (Pieterse et al., 2009). This has been observed in *amt1.1* plants upon *P. syringae* infection. In fact, SA, JA, and JA-Ile remain lower compared to wild type plants during a time-course experiment. Therefore, the reduced susceptibility of *amt1.1* to the bacterium cannot be caused by changes in the hormonal balance. Except for IAA, the hormones tested remained at lower levels in the absence of infection. This suggests that disruption of *AMT1.1* affects the basal levels of SA, JA, and JA-Ile, producing several constitutive changes. Interestingly, these basal changes are not visible in 2-week old plants; therefore, some alterations affected by the mutation are age dependent. For non-host resistance, such as for *P. cucumerina*, the defense responses are horizontal, multigenic and not controlled by single hormonal pathway, because other hormones in addition to SA, JA, ABA, and ET influence *Arabidopsis* responses against this necrotroph (Sanchez-Vallet et al., 2010). These compounds include the β-subunit of the heterotrimeric G-protein (Delgado-Cerezo et al., 2012) and glucosinolates and other Trp derivatives (Sánchez-Vallet et al., 2012; Gamir et al., 2014). In our first approach, we showed that *amt1.1* influences the SA, JA-Ile, and IAA profile during infection. Both JA-Ile and IAA over-accumulate, whereas SA is at lower levels compared to *Col3-gl1*. This may partially explain the observed phenotype because *P. cucumerina* has a necrotrophic lifestyle and is sensitive to JA-dependent signaling (Thomma et al., 2000). Recent research has suggested complex interplay between hormones in addition to the negative crosstalk between SA and JA (Pieterse et al., 2009), IAA also has antagonistic effects with SA, and both may explain the lower amounts of SA observed in *amt1.1*, although the final link between the ammonium transporter and hormonal signaling remains to be elucidated. *Arabidopsis* can also resist *P. cucumerina* through two other major mechanisms, callose deposition and camalexin (Ton and Mauch-Mani, 2004; García-Andrade et al., 2011; Sánchez-Vallet et al., 2012). Both mechanisms are directly or indirectly influenced by *AMT1.1*. Early callose deposition is enhanced in the mutant, and the camalexin levels remain higher at 48 hpi compared to *Col3-gl1*.

In our *Arabidopsis*-*P. syringae* system, we observed that *amt1.1* does not influence the basal amino acid accumulation in response to the bacterium. By contrast, there is a significant change in the amino acid profile for *P. cucumerina*. The mutant *amt1.1* over-accumulates several amino acids in the absence of infection, but these changes are even more robust after infection. Up to 11 amino acids over-accumulate in *amt1.1* infected plants compared to *Col3-gl1*. Surprisingly, Trp remains at the same level as the wild type plants with or without *P. cucumerina*. This may be explained by the over-accumulation of other Trp derivatives, such as indolic glucosinolates, IAA, camalexin, and other indole conjugates (Figure 9). These compounds are metabolic sinks that may trap the putative overproduction of Trp.

According to the PCA, the distance between *Col3-gl1* and *amt1.1* upon infection by both pathogens is rather marked; however, the diversity of the metabolic responses against *P. cucumerina* is much wider compared with the bacterium. In fact, of all of the compounds analyzed by targeted and untargeted chromatography that were found in clusters of over-accumulated metabolites in *amt1.1* upon *P. syringae* infection, only five pathways were identified, and of 45 signals, 24 were tentatively assigned to the phenylpropanoids (Table 1) and 5 to benzoic acid derivatives. This suggests that shikimic acid derivatives may compete with SA accumulation, which is a likely explanation for the reduced susceptibility of *amt1.1* and the reduced levels of free SA. Phenylpropanoids have been associated with plant resistance against a wide range of pathogens (Shadle et al., 2003). Two other compounds over-accumulated in amt1.1 plants are vitamins B1 and B6. Both have been linked to disease resistance (Denslow et al., 2005; Ahn et al., 2007). Exogenous treatment with vitamin B1 can induce defense priming in *Arabidopsis* against *P. syringae* mediated by H_2_O_2_ accumulation, callose deposition, and *NPR1*. Vitamin B6 is involved in superoxide quenching and stress responses, and during *P. syringae* infection in *Nicotiana*, vitamin B6 acts as an antioxidant and modulator of active oxygen species (Denslow et al., 2005). The relevance of the other metabolites, such as small peptides, nucleotides and CoA conjugates in the defense against pathogens must be clarified. Of the signals over-accumulated in *amt1.1* infected with *P. cucumerina*, 51 were tentatively or fully identified. These compounds were mainly involved in the indolic and aliphatic glucosinolates pathways, Trp pathway, flavonoids, nucleotides, and fatty acids. Notably, glucosinolates have been directly linked to resistance against *P. cucumerina* Sánchez-Vallet et al., 2012; Gamir et al., 2014, and also callose deposition in response to PAMPs (Clay et al., 2009). Although no direct evidence for *AMT1.1* and glucosinolates has been previously reported, this may be worth investigating. The NRT/PTR transporters have been associated with glucosinolate translocation in *Arabidopsis*, mainly aliphatic (Nour-Eldin et al., 2012). Additionally, Met is a precursor of aliphatic glucosinolates (Kraker and Gershenzon, 2011). This amino acid highly accumulates in amt1.1 plants infected with *P. cucumerina*. Furthermore, three precursors of aliphatic glucosinolates are also elevated in *amt1.1* (Figure 9). These findings suggest a repressive influence of AMT1.1 on plant defenses against necrotrophic infection that result in changes in amino acid metabolism and/or peptide transport that causes increased defenses, most likely mediated by aliphatic and indolic glucosinolates. Other relevant metabolic contributions from different pathways may also be involved, such as chlorogenic acid, ABA, spermidine, several fatty acids, and other unclassified compounds that are also over-accumulated in *amt1.1* plants upon infection.

We previously demonstrated that the member of the high-affinity transport system of nitrate, the *NRT2.1* gene, in addition to transporting nitrate, is involved in the coordination of plant defense responses (Camañes et al., 2012b). This gene is de-regulated upon low nitrate concentrations in the roots, subsequently, the HATS is activated, providing tolerance mechanism against abiotic stresses (Gansel et al., 2001). Despite these functions, *NRT2.1* represses plant responses against bacterial and fungal pathogens. The metabolic interplay between nitrate uptake and resistance against pathogens is being elucidated. Because this resistance mechanism is multicomponent and non-pathogen-specific, the disruption of *NRT2.1* results in enhanced resistance against *P. syringae* and *P. cucumerina* through different defense mechanisms (Camañes et al., 2012b; Gamir et al., 2014).

Here, we demonstrate that *AMT1.1* acts as a negative regulator of *Arabidopsis* defense. Its disruption has a moderate impact on metabolomic changes upon *P. syringae* infection, which is correlated with a reduced susceptibility to the bacterium. By contrast, the metabolomic changes of *amt1.1* upon *P. cucumerina* challenge are significant, targeting Trp-derivatives and indolic glucosinolates. These changes may be responsible for the enhanced resistance of *amt1.1* against the fungus. This study and previous findings from our laboratory indicate that N transporters are transceptors that impact the transcriptome (Camañes et al., 2012b) and metabolome of *Arabidopsis* (Gamir et al., 2012, 2014) to coordinate abiotic stress tolerance and the negative regulation of biotic stress responses. The manner in which *NRT2.1* and *AMT1.1* regulate stress signaling remains to be identified; however, it is clear that the plant has mechanisms to distinguish and regulate different sources of stress. Transceptors appear to play a key role in integrating the environmental signals and plant defense responses.

### Conflict of interest statement

The authors declare that the research was conducted in the absence of any commercial or financial relationships that could be construed as a potential conflict of interest.

## Supplementary material

Figure S1 **Heat map analysis performed with Marvis (Filter and Cluster packages)**. Five and two week old plants either mock (*Col3-gl/1* and *amt1.1*) or either *P. syringae Col3-gl/1 P. syringae* and *amt1.1 P. syringae* or *P. cucumerina* inoculated (*Col3-gl/1 Pc* and *amt1.1 Pc)* plants were processed for relative quantification analysis by HPLC-QTOFMS data. The concentration of the metabolites was determined in all the samples by normalizing the chromatographic area for each compound with the dry weight of the corresponding sample. Heatmaps are generated by using Mar-Vis Filter and Cluster following a Kruskal-Wallys test (*p* < 0.01). Clusters overepresented *amt1.1* mock or infected (intense red colors) compared with wild type plants were selected for subsequent data analysis.

Figure S2 **Profile of additional hits in the overaccumulated compounds upon *P. cucumerina* infection in *amt1.1* compared with *Col3-gl1***. For experimental details see Figures 1, 9. The concentration of the metabolites was determined in all the samples by normalizing the chromatographic area for each compound with the dry weight of the corresponding sample. The compounds were tentatively identified using the exact mass criteria using the METLIN and Massbank databases. Those compounds tentatively identified have the exact mass indicated. Te compounds fully identified have no reference to the mass. The compounds were grouped by metabolic pathways according to KEGG and AraCyc databases. White bars are mock inoculated and filled bars are *P. cucmerina* infected plants. Leaf material from 150 individual plants were pooled together for each treatment × genotype combination. Boxplots represent average three independent experiments with two technical replicates. Only data showing a *p*-value below 0.01 after a Kuskal wallys test were used for pathway assignation (*n* = 6).
