# Supplementary figures and images for: Disruption of the ammonium transporter AMT1.1 alters basal defenses generating resistance against Pseudomonas syringae and Plectosphaerella cucumerina
Source: Front Plant Sci. 2014 May 30;5:231. doi: 10.3389/fpls.2014.00231 (PMC4038795; doi:10.3389/fpls.2014.00231)

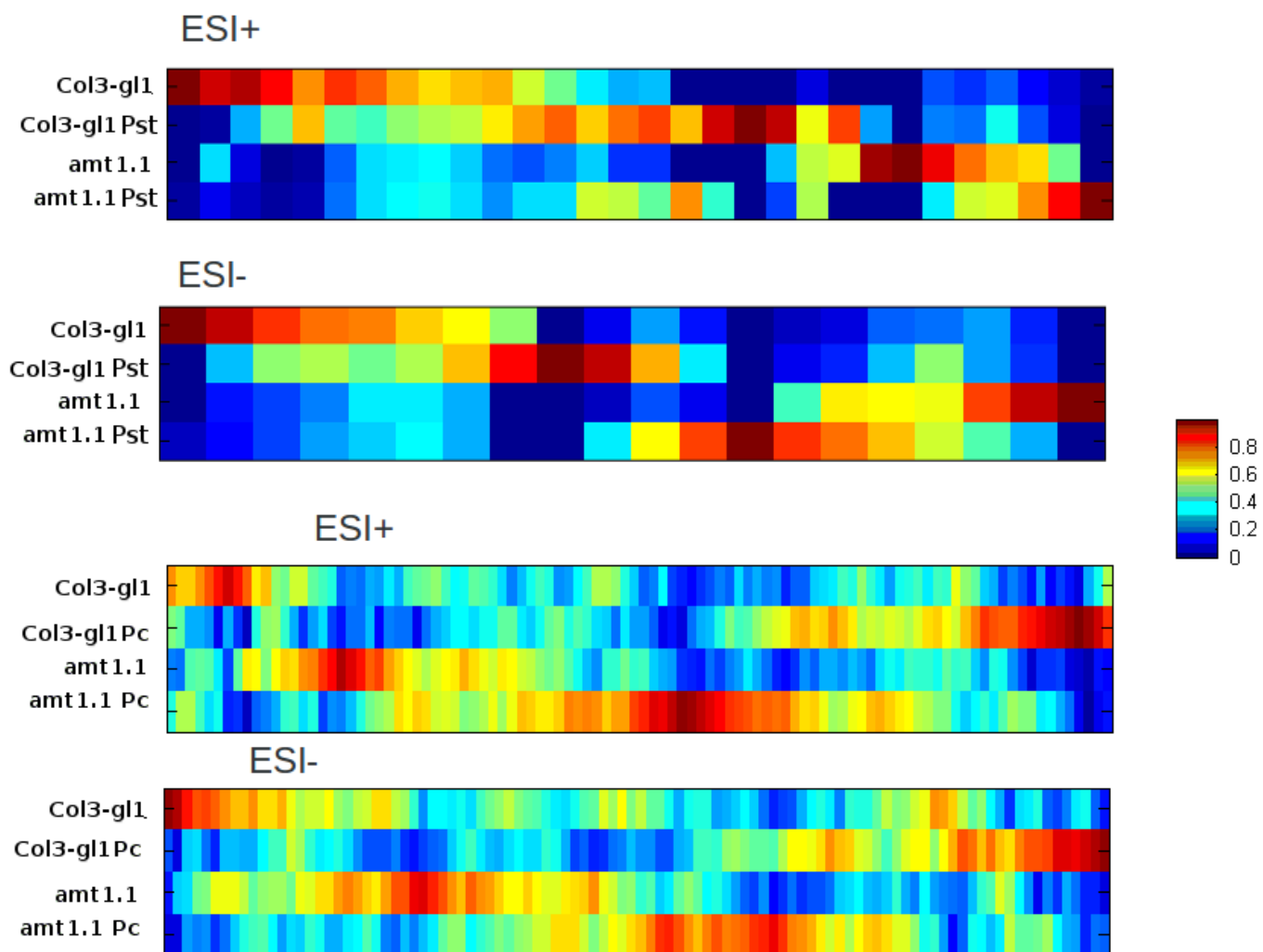

Figure S1

## Fatty acids pathway

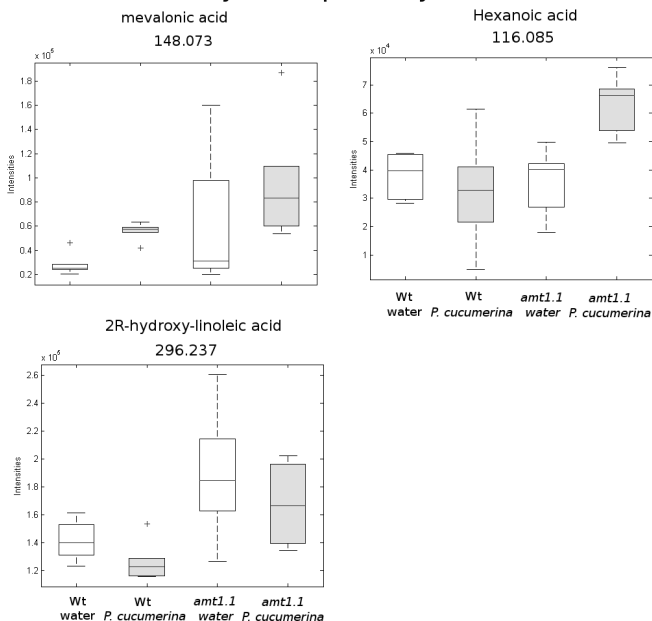

## Nucleotides

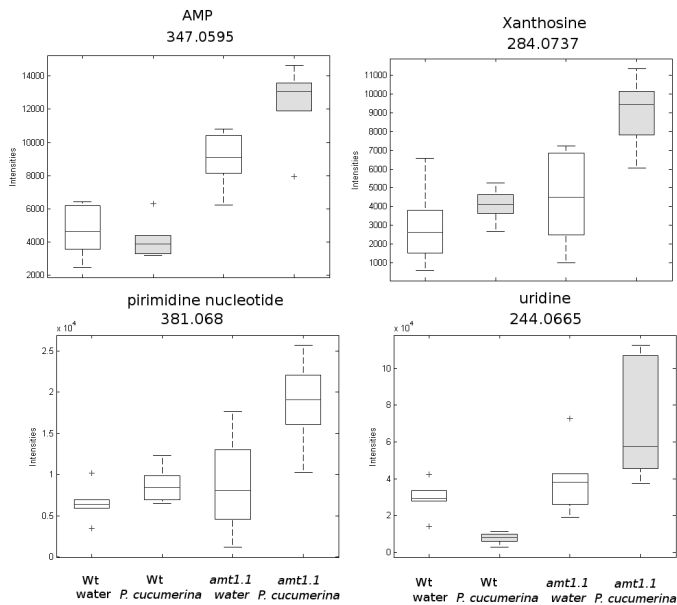

## Unclassified pathways

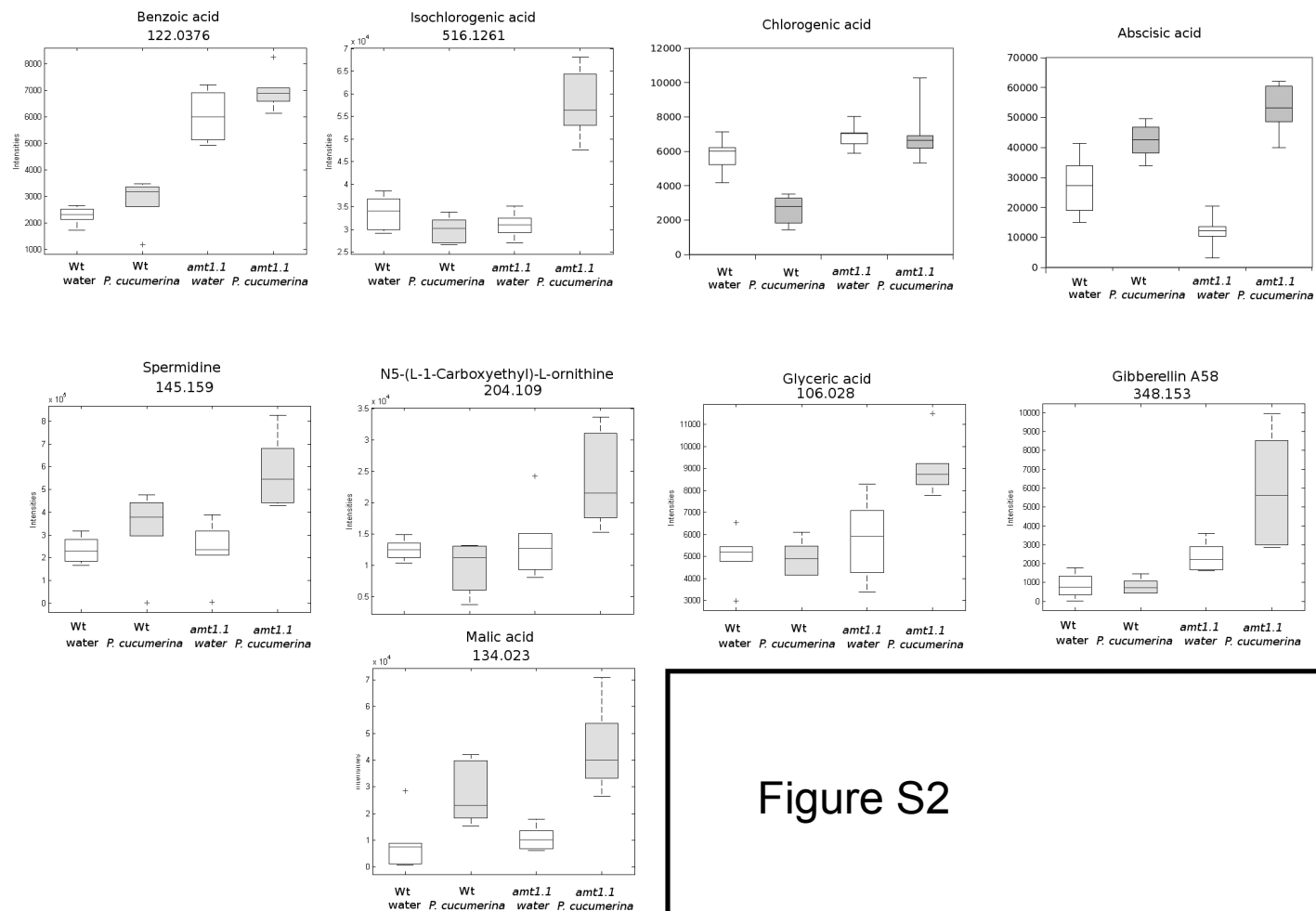

Figure S2

Supplement: Figure S1 — Heat map analysis performed with Marvis (Filter and Cluster packages). Five and two week old plants either mock (Col3-gl/1 and amt1.1) or either P. syringae Col3-gl/1 P. syringae and amt1.1 P. syringae or P. cucumerina inoculated (Col3-gl/1 Pc and amt1.1 Pc) plants were processed for relative quantification analysis by HPLC-QTOFMS data. The concentration of the metabolites was determined in all the samples by normalizing the chromatographic area for each compound with the dry weight of the corresponding sample. Heatmaps are generated by using Mar-Vis Filter and Cluster following a Kruskal-Wallys test (p < 0.01). Clusters overepresented amt1.1 mock or infected (intense red colors) compared with wild type plants were selected for subsequent data analysis. [file Presentation1.PDF]
